# Use of a wearable accelerometer to evaluate physical frailty in people receiving haemodialysis

**DOI:** 10.1186/s12882-023-03143-z

**Published:** 2023-03-31

**Authors:** Tobia Zanotto, Thomas H. Mercer, Marietta L. van der Linden, Jamie P. Traynor, Pelagia Koufaki

**Affiliations:** 1 Department of Occupational Therapy Education, School of Health Professions, University of Kansas Medical Center, 3901 Rainbow Blvd, Kansas City, KS 66160 USA; 2 Mobility Core, University of Kansas Center for Community Access, Rehabilitation Research, Education and Service, Kansas City, KS USA; 3 Centre for Health, Activity and Rehabilitation Research, School of Health Sciences, Queen Margaret University, Edinburgh, UK; 4 Renal and Transplant Unit, Queen Elizabeth University Hospital, Glasgow, UK

**Keywords:** Chronic kidney disease, Haemodialysis, Frailty, Accelerometer, Physical activity

## Abstract

**Background:**

Physical frailty is a major health concern among people receiving haemodialysis (HD) for stage-5 chronic kidney disease (CKD-5). Wearable accelerometers are increasingly being recommended to objectively monitor activity levels in CKD-5 and recent research suggests they may also represent an innovative strategy to evaluate physical frailty in vulnerable populations. However, no study has yet explored whether wearable accelerometers may be utilised to assess frailty in the context of CKD-5-HD. Therefore, we aimed to examine the diagnostic performance of a research-grade wearable accelerometer in evaluating physical frailty in people receiving HD.

**Methods:**

Fifty-nine people receiving maintenance HD [age = 62.3 years (SD = 14.9), 40.7% female] participated in this cross-sectional study. Participants wore a uniaxial accelerometer (ActivPAL) for seven consecutive days and the following measures were recorded: total number of daily steps and sit-to-stand transitions, number of daily steps walked with cadence < 60 steps/min, 60–79 steps/min, 80–99 steps/min, 100–119 steps/min, and ≥ 120 steps/min. The Fried phenotype was used to evaluate physical frailty. Receiver operating characteristics (ROC) analyses were performed to examine the diagnostic accuracy of the accelerometer-derived measures in detecting physical frailty status.

**Results:**

Participants classified as frail (*n* = 22, 37.3%) had a lower number of daily steps (2363 ± 1525 vs 3585 ± 1765, *p* = 0.009), daily sit-to-stand transitions (31.8 ± 10.3 vs 40.6 ± 12.1, *p* = 0.006), and lower number of steps walked with cadence of 100–119 steps/min (336 ± 486 vs 983 ± 797, *p* < 0.001) compared to their non-frail counterparts. In ROC analysis, the number of daily steps walked with cadence ≥ 100 steps/min exhibited the highest diagnostic performance (AUC = 0.80, 95% CI: 0.68–0.92, *p* < 0.001, cut-off ≤ 288 steps, sensitivity = 73%, specificity = 76%, PPV = 0.64, NPV = 0.82, accuracy = 75%) in detecting physical frailty.

**Conclusions:**

This study provided initial evidence that a wearable accelerometer may be a useful tool in evaluating physical frailty in people receiving HD. While the total number of daily steps and sit-to-stand transitions could significantly discriminate frailty status, the number of daily steps walked with cadences reflecting moderate to vigorous intensity of walking may be more useful in monitoring physical frailty in people receiving HD.

## Background

Low levels of physical activity have consistently been linked to lower quality of life, hospitalisations and increased mortality in people receiving haemodialysis (HD) for stage-5 chronic kidney disease (CKD-5) [1]. While patient-reported physical activity questionnaires are expedient, they have recognised limitations and more objective instruments, such as wearable accelerometers, are increasingly being recommended to accurately monitor activity levels and free-living ambulation in this clinical population [2]. Accelerometer-derived measures provide an increasingly excellent evidence base in terms of predicting adverse outcomes [3], and a growing number of studies have recently sought to develop stringent methodological criteria by recommending specific minimum wear-time of various accelerometers in dialysis populations [4, 5]. This would seem to suggest that accelerometers are gaining momentum in the context of CKD-5-HD and they may be shortly used as part of routine care (as both measurement tools for practitioners and physical activity promotional devices for patients) in dialysis units. In addition to providing high-quality information on physical activity behaviours and clinically relevant aspects of free-living ambulation (e.g., number of daily steps, step cadence, etc.), accelerometers may represent a viable strategy to evaluate physical frailty in vulnerable populations [6–8]. Indeed, two recent systematic reviews have concluded that several walking-related measures collected via wearable sensors can significantly discriminate frailty status in community-dwelling older adults [9, 10].

Physical frailty is a major health concern among people living with CKD-5 and upwards of one third of people receiving HD meet objective diagnostic criteria for frailty [11]. This biological syndrome has been linked to multiple adverse clinical outcomes in dialysis populations including, but not limited to, falls, fractures, lower access to kidney transplantation and increased mortality [12]. Consequently, there is a critical need to identify easily implementable and low-cost strategies to evaluate the presence and the trajectory of frailty in individuals receiving HD therapy, as this would lead to better clinical decision making [13]. In this respect, it has been recently proposed that use of remote sensor technology, such as wearable accelerometers, may improve the ability to recognize signs of frailty early on in the context of chronic diseases [14]. Particularly, accelerometers can detect subtle modifications of walking performance and physical activity levels that may reflect fine-grained changes in physiological function along the fit-to-frail continuum [6]. In addition, accelerometers have the advantage of measuring physical behaviour in a free-living environment, which could translate into a more ecologically valid assessment of frailty [8]. To date, however, no studies have yet explicitly explored the potential utility of using wearable technology to assess and monitor frailty levels in CKD-5-HD populations.

Therefore, the purpose of this investigation was to explore the diagnostic performance of a research-grade wearable accelerometer (ActivPAL) in evaluating physical frailty in people receiving HD for CKD-5. Our aims were to 1) characterise objective levels of physical activity, collected with the accelerometer (i.e., in free-living conditions), in frail and non-frail people living with CKD-5 and receiving HD, and 2) to examine the diagnostic accuracy of the wearable accelerometer in evaluating frailty within the same study population. We hypothesised that frail individuals would have a lower number of daily steps and sit-to-stand transitions compared to the non-frail, and that accelerometer-based measures of physical activity would be able to significantly discriminate frailty status in people receiving HD.

## Methods

### Study design and participants

This study consisted of a secondary analysis of cross-sectional accelerometer data from a multicentre observational study on frailty and falls in CKD-5-HD (NCT02392299). Participants were people aged 18 years or older (both men and women), able to comprehend written and spoken English, and receiving maintenance HD thrice weekly in a Renal Unit based in the UK. Exclusion criteria for the study were: unstable dialysis and cardiovascular conditions (e.g., suspected or known aneurysm, critical cerebrovascular stenosis, critical proximal coronary artery stenosis, critical mitral stenosis, clinically severe left ventricular outflow obstruction), lower limb amputation without prosthesis, and severe cognitive impairment. The study protocol was reviewed and approved by the Queen Margaret University research ethics committee and by the local National Health Service research ethics committee (15/WS/0079) and conformed to the ethical standards for medical research involving human subjects, as laid out in the 1964 Declaration of Helsinki and its later amendments. Participants provided written informed consent prior to taking part in the study.

### Procedures

All study procedures were performed by a researcher highly experienced in frailty evaluations during a single participant assessment visit at the Renal Unit and were conducted on a non-HD day. Participants were provided with an ActivPAL accelerometer (PAL Technologies Ltd, Glasgow, UK) as part of a multidimensional assessment of physical function [15]. Participants were instructed to wear the accelerometer on the anterior aspect of the thigh for seven consecutive days, during waking hours, and to report the wear time daily using an activity log. The ActivPAL is a uniaxial accelerometer that uses software-derived algorithms to measure number of steps and sit-to-stand transitions, as well as time spent in different postures from thigh inclination, with a sampling frequency of 10 Hz. ActivPAL data were inspected for monitor malfunctions through the PAL Technologies software and were exported to an Excel spreadsheet to enable accurate determination of accelerometer wear time. Participants were excluded from the analysis if they had less than eight hours per day of wear time and if they wore the accelerometer for less than three days (two dialysis and one non-dialysis), as recommended by previous research [5]. Additionally, the first day of accelerometer wear was considered a ‘habituation’ period for participants and was therefore discarded from the final analysis. The following ActivPAL measures were taken for analysis: number of daily steps, number of daily sit-to-stand transfers, and number of daily steps walked with cadences < 60 steps/min, 60–79 steps/min, 80–99 steps/min, 100–119 steps/min, and ≥ 120 steps/min [16]. In addition, as a secondary measure, we also calculated the percentage of daily steps that were walked with the step cadences described above to account for differences in the total number of daily steps between the two groups (i.e., frail vs non-frail).

A modified version of the Fried phenotype was used to assess physical frailty [17]. The exact operationalisation of the frailty definition used in the current study is fully summarised in Table 1. Participants were classified as frail if they met at least three out of five components of the modified Fried phenotype (i.e., slow walking speed, exhaustion, low physical activity, weakness, unintended weight loss), as described elsewhere [15]. Demographic (e.g., age, body mass, height) and clinical characteristics (e.g., dialysis vintage, medications, biochemistry values) of the study participants were extracted from their medical records. Biochemistry values were collected as part of monthly routine visits in the Renal Unit, as close as possible to the study visit (within one month).

**Table 1:** Operationalisation of frailty used in the current study (modified Fried criteria)

**Frailty components**	**Fried phenotype criteria**		**Modified Fried criteria**	
1. Low gait speed (slowness)	Time to walk 15 feet (4.57 m) above a cut-off value stratified by gender and height:		Time to walk 15 feet (4.57 m) above a cut-off value stratified by gender and height (same criteria used by Fried et al. [17])	
	*Men*	*Cut-off*		
	Height ≤ 173 cm	≥ 7 s		
	Height > 173 cm	≥ 6 s		
	*Women*			
	Height ≤ 159 cm	≥ 7 s		
	Height > 159 cm	≥ 6 s		
2. Low muscle strength (weakness)	Isometric handgrip test below a cut-off value stratified by gender and BMI:		Isometric handgrip test below a cut-off value stratified by gender and BMI (same criteria used by Fried et al. [17])	
	*Men*	*Cut-off*		
	BMI ≤ 24	≤ 29 kg		
	BMI: 24.1 – 26	≤ 30 kg		
	BMI: 26.1 – 28	≤ 30 kg		
	BMI > 28	≤ 32 kg		
	*Women*			
	BMI ≤ 23	≤ 17 kg		
	BMI: 23.1 – 26	≤ 17.3 kg		
	BMI: 26.1 – 29	≤ 18 kg		
	BMI > 29	≤ 21 kg		
3. Low physical activity (inactivity)	Kcal/week of physical activity below a cut-off value stratified by gender (calculated using the standardised algorithm of the Short-Form Minnesota Leisure Time Activity Questionnaire):		Kcal/week of physical activity below a cut-off value stratified by gender (calculated using the standardised algorithm of the Short-Form International Physical Activity Questionnaire):	
	*Men*	< 383 kcal/week	*Men*	< 383 kcal/week
	*Women*	< 270 kcal/week	*Women*	< 270 kcal/week
4. Poor endurance (exhaustion)	Answering ‘a moderate amount of the time’ or ‘most of the time’ to the following two statements from the CES-D questionnaire: 1) ‘I felt that everything I did was an effort’, 2) ‘I could not get going’		Vitality score < 55 using the SF-36 questionnaire [25]	
5. Weight loss (shrinkage)	Unintended weight loss ≥ 10 lbs (4.54 kg) in the previous 12 months		Unintended weight loss ≥ 10 lbs (4.54 kg) in the previous 12 months (same criteria used by Fried et al. [17])	

*BMI* Body mass index, *CES-D* Center for Epidemiological Studies–Depression scale, *SF-36* 36-Item Short Form Health Survey

### Statistical analysis

Statistical analyses were performed with SPSS, Version 27.0 (IBM, Inc., Armonk, NY). The Kolmogorov–Smirnov test was used to check whether data were normally distributed. Differences between frail and non-frail participants in demographics, clinical characteristics and accelerometer-derived measures were analysed by means of a Chi-Squared test for categorical variables, and through independent t-tests or Mann–Whitney U tests, as appropriate, for continuous variables. The diagnostic accuracy of ActivPAL measures to detect physical frailty (yes/no) was explored through receiver operating characteristics (ROC) analysis. Classifier evaluation metrics included the area under the curve (AUC), the K-S statistic, and test cut-offs along with their sensitivity and specificity. The positive and negative predictive (PPV and NPV) values and total accuracy were also determined. In a sensitivity analysis, we performed an additional ROC analysis using the ActivPAL measures normalised by daily wear time to account for the potential confounding effect of discretionary accelerometer wear by the study participants. A significance level of *p <* 0.05 was used to guide the statistical interpretation of all the performed analyses.

## Results

The data of 76 participants who were provided with the wearable accelerometer were studied in the current analysis. However, 17 participants were excluded as they did not achieve the minimum required accelerometer wear time. Therefore, the data of 59 participants were included in the final analysis. Participants (59.3% male, 40.7% female) had a mean age of 62.3 years (SD = 14.9) with measured body mass index of 28.3 kg*m^−2^ (SD = 5.6), albumin = 37.1 g/L (SD = 4.2), creatinine = 619.3 umol/L (SD = 145.3), haemoglobin = 11.2 g/dL (SD = 1.1), parathyroid hormone = 28.0 pmol/L (SD = 34.8), and urea reduction ratio = 71.1% (SD = 5.6). Participants had a median dialysis vintage of 1.1 years (IQR = 2.2) and were prescribed with a median of 11.0 medications (IQR = 5.0). Twenty-two (37.3%) participants met three or more frailty criteria and were therefore classified as frail, while the remaining 37 (62.7%) participants were classified as non-frail. Among the non-frail, 31 participants met one or two frailty criteria, which are commonly used to indicate a pre-frailty status [6, 7]. On the other hand, only six participants did not exhibit any component of frailty (Table 2). The differences in demographic and clinical characteristics between frail and non-frail participants are reported in Table 3. Table 4 summarises all ActivPAL data in the study population. Compared to their non-frail counterparts, frail participants had a lower number of daily steps, daily sit-to-stand transitions, and lower number of steps walked with cadence of 100–119 steps/min. Additionally, frail participants also had a higher percentage of steps walked with step cadences  < 80 steps/min and a lower percentage of steps walked with step cadences ≥ 100 steps/min, compared to the non-frail.

**Table 2:** Frailty components in the study population

**Frailty components**	**Non-frail (** ***n*** ** = 37)**		**Frail (** ***n*** ** = 22)**
	Robust (*n* = 6)	Pre-frail (*n* = 31)	
Low gait speed (slowness)	n/a	2 (6.5%)	16 (72.7%)
Low muscle strength (weakness)	n/a	9 (29.0%)	19 (86.4%)
Low physical activity (inactivity)	n/a	14 (45.2%)	18 (81.8%)
Poor endurance (exhaustion)	n/a	22 (71.0%)	21 (95.5%)
Weight loss (shrinkage)	n/a	3 (9.7%)	8 (36.4%)

*Abbreviations: n/a* not applicable; “Non-frail” indicates participant meeting ˂3 frailty components; “Robust” indicates participant meeting 0 frailty components; “Pre-frail” indicates participant meeting 1–2 frailty components; “Frail” indicates participant meeting ≥ 3 frailty components

**Table 3:** Demographics and clinical characteristics: differences between frail and non-frail participants. Results are expressed as mean ± SD or median [IQR]

Variables	Frail (22)	Non-frail (37)	*P*-value
Age (years)	68.6 ± 9.1	58.5 ± 16.4	0.004
Gender, F (n, %)	9(40.9)	15(40.5)	0.978
BMI (kg*m^−2^)	28.3 ± 6.2	28.3 ± 5.2	0.986
Albumin (g/L)	35.8 ± 4.0	37.8 ± 4.2	0.074
Creatinine (umol/L)	553.4 ± 127.5	658.5 ± 142.5	0.006
Hb (g/dL)	11.2 ± 1.0	11.2 ± 1.2	0.959
PTH (pmol/L)	33.1 ± 46.4	24.9 ± 25.8	0.385
URR (%)	71.4 ± 6.4	70.9 ± 5.2	0.773
Dialysis vintage (years)	1.2[2.1]	1.0[2.3]	0.481
Prescribed medications (n)	12.5[6.5]	10.0[3.0]	0.030

*Abbreviations: BMI* Body mass index, *Hb* Haemoglobin, *IQR* Interquartile range, *PTH* Parathyroid hormone, *SD* Standard deviation, *URR* Urea reduction ratio

**Table 4:** ActivPAL data in the study population: differences between frail and non-frail participants. Results are expressed as mean ± SD

Variables	Frail (22)	Non-frail (37)	*P*-value
Daily steps (n°)	2363 ± 1525	3585 ± 1765	0.009
Daily sit to stands (n°)	31.8 ± 10.3	40.6 ± 12.1	0.006
Step cadence < 60 s/min			
Number of steps (n°)	547 ± 327	624 ± 276	0.337
Percentage of daily steps (%)	29.3 ± 18.0	18.9 ± 6.3	0.016
Step cadence 60–79 s/min (n° steps)			
Number of steps (n°)	572 ± 387	595 ± 248	0.778
Percentage of daily steps (%)	26.3 ± 13.6	18.2 ± 6.3	0.015
Step cadence 80–99 s/min (n° steps)			
Number of steps (n°)	877 ± 682	1175 ± 623	0.091
Percentage of daily steps (%)	32.4 ± 16.1	34.2 ± 9.7	0.634
Step cadence 100–119 s/min (n° steps)			
Number of steps (n°)	336 ± 486	983 ± 797	< 0.001
Percentage of daily steps (%)	11.0 ± 13.0	24.2 ± 12.6	< 0.001
Step cadence ≥ 100 s/min (n° steps)			
Number of steps (n°)	383 ± 563	1277 ± 1389	< 0.001
Percentage of daily steps (%)	12.7 ± 15.8	29.9 ± 19.7	< 0.001
Step cadence ≥ 120 s/min (n° steps)			
Number of steps (n°)	46 ± 89	294 ± 914	0.213
Percentage of daily steps (%)	1.7 ± 3.3	5.7 ± 12.6	0.150

*Abbreviations: SD* Standard deviation; Percentage of daily steps represents the ratio between the number of daily steps walked at a specific cadence and the total number of daily steps (expressed as a percentage)

**Fig. 1:**
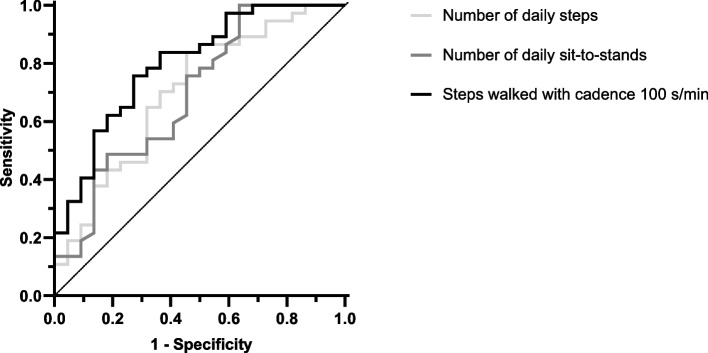
ROC analysis: ROC curves of daily steps, daily sit-to-stands, and number of daily steps walked with cadence ≥ 100 steps/min Legend: ROC: receiver operating characteristics

The results of the ROC analysis are summarised in Table 5. The following variables were statistically significant discriminators of frailty status: number of daily steps (AUC = 0.70, *p* = 0.005), number of daily sit-to-stand transfers (AUC = 0.70, *p* = 0.008), number of daily steps walked with cadence 100–119 steps/min (AUC = 0.79, *p* ˂ 0.001) and with cadence ≥ 120 steps/min (AUC = 0.74, p ˂ 0.001). Due to the very low number of steps walked with cadence ≥ 120 steps/min (Table 4), we calculated the additional variable ‘number of daily steps walked with cadence ≥ 100 steps/min’. This variable exhibited the highest diagnostic performance in ROC analysis (AUC = 0.80, 95% CI: 0.68—0.92, *p* < 0.001, cut-off ≤ 288 steps, sensitivity = 73%, specificity = 76%, PPV = 0.64, NPV = 0.82, accuracy = 75%). Figure 1 displays the ROC curve of this last variable in comparison with the ROC curves of total number of daily steps and sit-to-stand-transitions. In addition, the number of daily steps walked with step cadences < 80 steps/min were also able to detect frailty status, when expressed as a percentage of the total daily steps (Table 5).

**Table 5:** ROC analysis of ActivPal measures for the assessment of physical frailty in people receiving haemodialysis

ActivPal measures	AUC (95% CI)	*P*-value	K-S	Cut-off	Prevalence, n (%)	SENS	SPEC	PPV	NPV	Accuracy
Daily steps (n°)	0.70 (0.56–0.84)	0.005	0.38	≤ 1980	18 (30.5)	55%	84%	0.67	0.76	73%
Daily sit to stands (n°)	0.70 (0.55–0.84)	0.008	0.36	≤ 26	10 (16.9)	36%	95%	0.80	0.71	73%
Step cadence < 60 s/min										
Number of steps (n°)	0.62 (0.47–0.78)	0.107	0.29	≥ 632	24 (40.7)	23%	49%	0.21	0.51	39%
Percentage of daily steps (%)	0.74 (0.60–0.87)	0.001	0.43	≥ 23.8	19 (32.2)	59%	84%	0.68	0.78	75%
Step cadence 60–79 s/min										
Number of steps (n°)	0.57 (0.41–0.72)	0.422	0.08	≥ 1001	5 (8.5)	14%	94%	0.60	0.65	64%
Percentage of daily steps (%)	0.72 (0.58–0.86)	0.003	0.46	≥ 22.8	18 (30.5)	59%	87%	0.72	0.78	76%
Step cadence 80–99 s/min										
Number of steps (n°)	0.64 (0.48–0.80)	0.093	0.34	≤ 694	17 (28.8)	50%	84%	0.65	0.74	71%
Percentage of daily steps (%)	0.51 (0.35–0.68)	0.872	0.18	≤ 13.5	4 (6.8)	18%	97%	0.80	0.67	69%
Step cadence 100–119 s/min										
Number of steps (n°)	0.79 (0.67–0.91)	< 0.001	0.48	≤ 284	25 (42.4)	73%	76%	0.64	0.82	75%
Percentage of daily steps (%)	0.78 (0.65–0.92)	< 0.001	0.53	≤ 18.3	26 (44.1)	77%	76%	0.65	0.85	76%
Step cadence ≥ 100 s/min										
Number of steps (n°)	0.80 (0.68–0.92)	< 0.001	0.48	≤ 288	25 (42.4)	73%	76%	0.64	0.82	75%
Percentage of daily steps (%)	0.78 (0.65–0.92)	< 0.001	0.53	≤ 19.1	26 (44.1)	77%	76%	0.65	0.85	76%
Step cadence ≥ 120 s/min										
Number of steps (n°)	0.74 (0.61–0.87)	< 0.001	0.48	≤ 15	28 (47.4)	77%	70%	0.61	0.84	73%
Percentage of daily steps (%)	0.72 (0.59–0.86)	0.001	0.44	≤ 0.5	24 (40.7)	68%	76%	0.63	0.79	71%

*Abbreviations: AUC* Area under the curve, *CI* Confidence interval, *K-S* KS statistic, *SENS* Sensitivity, *SPEC* Specificity, *PPV* Positive predictive value, *NPV* Negative predictive value; Percentage of daily steps represents the ratio between the number of daily steps walked at a specific cadence and the total number of daily steps (expressed as a percentage)

Table 6 summarises the sensitivity ROC analysis performed on the accelerometer measures normalised by daily wear time. This analysis yielded similar results, as the number of daily steps (AUC = 0.67, *p* = 0.022), the number of daily sit-to-stand transfers (AUC = 0.65, *p* = 0.040), and the number of daily steps walked with cadences of 100–119 steps/min (AUC = 0.78, *p < *0.001), ≥ 100 steps/min (AUC = 0.77, *p < *0.001), and ≥ 120 steps/min (AUC = 0.73, *p* = 0.001) were still able to significantly discriminate frailty status.

**Table 6:** ROC analysis of ActivPal measures (normalised by wear time) for the assessment of physical frailty in people receiving haemodialysis

ActivPal measures	AUC (95% CI)	*P*-value	K-S	Cut-off	Prevalence, n (%)	SENS	SPEC	PPV	NPV	Accuracy
Daily steps/h (n°)	0.67 (0.53–0.82)	0.022	0.41	≤ 173.5	26 (45.6)	71.4%	69.4%	0.58	0.81	70.2%
Daily sit to stands/h (n°)	0.65 (0.51–0.80)	0.040	0.33	≤ 2.0	20 (35.1)	52.4%	75.0%	0.55	0.73	66.7%
Time spent sitting/lying (%)	0.48 (0.31–0.64)	0.773	0.08	≥ 71.05	49 (86.0)	81.0%	11.1%	0.35	0.50	36.8%
Time spent standing/stepping (%)	0.52 (0.36–0.69)	0.773	0.13	≤ 18.73	28 (49.1)	57.1%	55.6%	0.43	0.69	56.1%
Step cadence < 60 s/min										
Number of steps/h (n°)	0.57 (0.41–0.72)	0.411	0.21	≥ 38.28	24 (42.1)	28.6%	50.0%	0.25	0.55	42.1%
Step cadence 60–79 s/min										
Number of steps/h (n°)	0.54 (0.38–0.70)	0.638	0.16	≤ 32.06	24 (42.1)	52.4%	63.9%	0.46	0.70	59.6%
Step cadence 80–99 s/min										
Number of steps/h (n°)	0.62 (0.46–0.78)	0.138	0.26	≤ 62.72	26 (45.6)	61.9%	63.9%	0.50	0.74	63.2%
Step cadence 100–119 s/min										
Number of steps/h (n°)	0.78 (0.65–0.90)	< 0.001	0.50	≤ 13.68	20 (35.1)	66.7%	83.3%	0.70	0.81	77.2%
Step cadence ≥ 100 s/min										
Number of steps/h (n°)	0.77 (0.63–0.90)	< 0.001	0.50	≤ 14.17	20 (35.1)	66.7%	83.3%	0.70	0.81	77.2%
Step cadence ≥ 120 s/min										
Number of steps/h (n°)	0.73 (0.59–0.87)	0.001	0.44	≤ 0.83	25 (43.9)	71.4%	72.2%	0.60	0.81	71.9%

*Abbreviations: AUC* Area under the curve, *CI* Confidence interval, *K-S* KS statistic, *SENS* Sensitivity, *SPEC* Specificity, *PPV* Positive predictive value, *NPV* Negative predictive value

## Discussion

The current study aimed to examine the diagnostic performance of a research-grade wearable accelerometer in evaluating physical frailty in a convenience sample of people living with CKD-5 and receiving HD. Our hypothesis that frail participants would have a lower number of daily steps and sit-to-stand transitions compared to their non-frail counterparts was confirmed by the analysis (Table 4). In addition to the total number of steps and sit-to-stand transitions, other measures of walking-related activity, such as the number of steps walked with step cadences ≥ 100 steps/min also exhibited a fair to good diagnostic accuracy (i.e., 0.73 ≤ AUCs ≤ 0.80) in detecting frailty status in the studied population (Tables 5 and 6).

The successful implementation of wearable accelerometers into routine renal care depends on their proven prognostic utility. This study provided initial evidence that ActivPAL accelerometers may be useful in aiding the evaluation of physical frailty in people receiving HD. In agreement with findings from two recent systematic reviews conducted in community-dwelling older adults [9, 10], the total number of daily steps and sit-to-stand transitions were fairly accurate in detecting frailty status in our cohort (Table 5). However, the number of daily steps walked with cadence ≥ 100 steps/min exhibited a better diagnostic performance (Fig. 1). Step cadence is an established domain of free-living ambulation and values of ≥ 100 steps/min have consistently been used to indicate moderate intensity of walking [16]. Importantly, monitoring the daily number of steps may represent a potentially useful outcome as previous research has proposed that increasing the total number of daily steps may attenuate frailty progression in elderly populations [18]. Aligned with our results, Pradeep Kumar et al., [7] have recently shown that daily step-counts can detect frailty status, as operationalised through the Fried phenotype, in community-dwelling older adults (AUC = 0.77). Nevertheless, in the context of CKD-5-HD, the interindividual variability of daily steps is often clamped by the prolonged periods of sedentary behaviour imposed by the HD treatment [5]. This may explain the lower diagnostic accuracy of total number of daily steps observed in our study (AUC = 0.70). On the other hand, accelerometer-derived metrics reflecting the ability to perform moderate to vigorous ambulation, such as step cadence ≥ 100 steps/min (AUC = 0.80), may be a more suitable choice for physical frailty evaluation in people receiving HD.

It should also be noted that, mirroring the observation on the lower number of steps walked with cadences ≥ 100 steps/min, frail participants also exhibited a higher percentage of steps walked with cadences < 80 steps/min (Tables 4 and 5). Particularly, frail individuals walked approximately 56% and 88% of their steps with cadences inferior to 80 steps/min, and  < 100 steps/min, respectively (Table 4). This observation further reinforces the notion that accelerometers can capture physical activity measures indicative of reduced physiological reserve (i.e., reduced capacity to engage in moderate to vigorous walking-related activities [19]), which in turn could aid health providers in evaluating the presence and/or changes in physical frailty.

In addition to allowing an objective and more accurate (compared to self-report tools) assessment of physical activity behaviour, a further benefit of using wearable accelerometers in people living with CKD-5 is that they also enable the evaluation of walking-related activity in real-life conditions or, in other words, beyond the clinic. Indeed, as previous research has shown, there can be significant differences in walking performance measures collected in a clinical environment as opposed to the real world [20]. In particular, walking tests performed in the clinic provide only a static snapshot of walking ability, and people may willingly or unwillingly modify their walking behaviour when they are observed. From this perspective, wearable accelerometers may increase the ecological validity of walking behaviour measurements [21]. Notably, recent advances in wearable technology have allowed the quantification of both the quantity (e.g.., number of daily steps, total amount of physical activity) and quality (e.g., gait speed, step cadence, gait variability) of walking performance [22, 23]. In this respect, the ActivPAL accelerometer can evaluate aspects of both walking quantity and quality. Particularly, the measure showing the highest diagnostic performance in our study (i.e., number of daily steps walked with cadence ≥ 100 steps/min) incorporates both aspects. This seems to open the possibility that combining accelerometer-based measures of walking quantity and quality may be a suitable strategy to evaluate physical frailty. In this regard, it should also be highlighted that a simple gait speed test performed in the clinic can detect physical frailty with an excellent diagnostic performance (AUC = 0.90) in people receiving HD [24]. Therefore, wearable technology capable of measuring an individual’s typical gait speed (i.e., in the real world) while providing additional information on several aspects of free-living walking performance may provide an accurate and more ecologically valid assessment of frailty in people living with CKD-5. While the current cross-sectional study suggests that wearable accelerometers may represent a viable strategy to assess physical frailty in a HD population, further studies with longitudinal design would be required to explore whether accelerometers can be used to validly monitor frailty status changes over time.

### Limitations

The findings form this study should be carefully interpreted due to some methodological limitations. First, we should acknowledge that several conceptualisations of frailty exist. In the current investigation, we limited the scope of our research aims to physical frailty by using the Fried phenotype [17]. Therefore, the study results should be considered in light of this caveat, as using other definitions of frailty (e.g., deficit accumulation model, clinical frailty scale, etc.) may have yielded different results. Moreover, the exhaustion frailty criterion was modified in our study, as we used the vitality score ( < 55) from the SF-36 questionnaire to characterize this component (Table 1). While this modification has been validated by previous research [25], the deviation from the original Fried phenotype [17] may be construed as a study limitation. However, the prevalence of frailty emerging from the study (i.e., 37.3%) is representative of the general population of people receiving HD, as indicated by previous meta-analyses [26]. This suggests that, despite the slight definitional modification, our operationalisation of frailty exhibits external validity. In addition to the considerations made for frailty, it should also be explicitly acknowledged that several research-grade wearable accelerometers are available on the market. Therefore, the observations made on the potential utility of wearables to evaluate physical frailty in CKD-5 are intended for the specific tool used in our study (i.e., ActivPAL) and may not be generalised to other wearable devices. Finally, it should be acknowledged that the sample size was relatively small and that, consequently, the inclusion of a larger sample would have enhanced the accuracy of diagnostic performance metrics such as sensitivity, specificity, PPV and NPV. Additionally, due to the relatively small sample size, we did not differentiate “robustness” from “pre-frailty” among non-frail participants. In this respect, further research would be required to examine whether wearable accelerometers may be useful in detecting the early stages of frailty in people receiving HD.

## Conclusions

The current study provided initial evidence that a wearable accelerometer (ActivPAL) may be useful in aiding the evaluation of physical frailty in people receiving HD for CKD-5. Frail participants performed a lower number of daily steps and sit-to-stand transitions compared to non-frail individuals, and these measures exhibited a fair diagnostic performance in discriminatory analyses. However, metrics that incorporated a component of ambulation intensity, such as the number of daily steps walked with cadences ≥ 100 steps/min were able to detect physical frailty status with a higher (i.e., good) diagnostic performance. Owing to the greater ecological validity of walking-related measures collected via wearable technology, findings from this study support the notion that wearable accelerometers may be clinically valuable to health care providers working in the dialysis unit, not only to objectively monitor physical activity levels but also to evaluate physical frailty and/or to track changes in frailty status.

## Data Availability

The datasets used and analysed during the current study are available from the corresponding author on reasonable request.

## Abbreviations

AUC: Area under the curve
CI: Confidence interval
CKD-5: Stage-5 chronic kidney disease
HD: Haemodialysis
IQR: Interquartile range
NPV: Negative predictive value
PPV: Positive predictive value
ROC: Receiver operating characteristics
SD: Standard deviation
